# Is There an Association Between Pseudoexfoliation and Pterygium? An Observational Study in a Semi-arid District of South India

**DOI:** 10.7759/cureus.38631

**Published:** 2023-05-06

**Authors:** Panaah Shetty, Inchara N

**Affiliations:** 1 Ophthalmology, Sri Devaraj Urs Academy of Higher Education and Research, Kolar, IND

**Keywords:** kolar, elderly population, agricultural workers, pex, pseudoexfoliation, pterygium

## Abstract

Introduction

Pseudoexfoliation (PXF) has a varied impact on the eye and has a complex relationship with pterygium and cataracts. We explored this study to estimate the proportion of PXF and its association with pterygium in cataract patients from a semi-arid district in South India.

Methods

This retrospective observational study was conducted at Sri Devaraj Urs Medical College, Sri Devaraj Urs Academy of Higher Education and Research, a tertiary care referral center in Kolar, India. Cataract patients attending the hospital between December 2020 and August 2022 were included based on non-probability sampling. Three hundred fifty-two patients were included based on the inclusion and exclusion criteria, and their records on demographic details and ocular examination were collected.

Results

Out of 352 patient records, 184 (52.27%) were males, with the mean age being 67.84 ± 13.08 years. In all, 95% of the patients were agricultural laborers exposed to sunlight and dust for more than six hours daily. It was observed that the proportion of PXF and pterygium in the study population was 28.40% (100) and 56.33% (199), respectively. The mean age of the PXF patients was 75.53 ± 6.26 years. The association of PXF with pterygium was statistically significant (p < 0.05).

Conclusion

PXF is one of the significant causes of complications in cataract surgery and blindness, which can be detected only in the end stages. This study draws a statistically significant association between pterygium and PXF. Identifying preclinical cases of PXF and halting its progression by avoiding risk factors such as prolonged exposure to sunlight, UV radiation, and dust should be focused on risk-prone geographical areas.

## Introduction

Pseudoexfoliation (PXF) syndrome is a generalized extracellular matrix disorder first described in 1917 by a Finnish Ophthalmologist. It is characterized by the production of abnormal basement membrane-like material in several intraocular and extraocular tissues. This abnormal fluffy white amyloid-like proteinaceous material gets deposited in the angle of the anterior chamber, trabecular meshwork, anterior surface of the iris, lens capsule, and cornea [1,2].

Previous population-based studies suggest that the prevalence of PXF globally ranges from 1.5% to 40%. Studies also show that in European countries, the proportion is 3.6-34.2%; in Africa, 1.5-40.9%; in Ethiopia, 12-13.2%; in Asian countries, 1.5-22.1%; and finally, in Southern India alone, 3.8% [3-8].

PXF has varied impacts on the eye, causing poor mydriasis, early cataract formation, weak zonular support, secondary glaucoma, and complications during cataract surgery [2,9]. Although it is one of the most commonly identifiable causes of open-angle glaucoma worldwide, very few studies exist globally, especially in India, to depict the prevalence of polyethylene (PEX) and understand the environmental factors that may cause this condition [1,3]. The trigger for the production of PEX material remains unidentified. However, various studies have suggested that certain genetic and environmental factors may lead to the development of PXF, like LOXL1 gene mutation, exposure to sunlight, viral infection, and trauma [4,10].

Few studies concluded that the UV rays in sunlight cause the development of PXF by upregulating the exfoliative material components. While the clinically visible exfoliative material is the diagnostic feature in manifest PXF, revelatory features of un-manifest or subclinical PXF are pupillary ruff abnormalities and reduced retinal nerve fiber layer (RNFL) thickness [9,11]. Also, there is electron microscopic evidence of these deposits on the other ocular structures, like conjunctiva, in subclinical cases of PXF. It is seen that pterygium shares a common etiology with PXF, like exposure to UV rays in sunlight, which, in addition to upregulation of exfoliative material, also causes alteration in limbal stem cells [11-13]. So, the presence of such external ocular signs, anterior segment findings, and common etiological factors can ensign the development of PXF in the subclinical or unmanifested eye. With this background, our study aims to find the proportion and association of pterygium in patients with PXF in a semi-arid district of south India.

## Materials and methods

Study design and setting

This is a retrospective observational study that was conducted at Sri Devaraj Urs Medical College, Sri Devaraj Urs Academy of Higher Education and Research, a tertiary care referral institute in Kolar, a semi-arid district of South India.

Inclusion criteria

Clinically significant PXF, subclinical PXF, and un-manifest PXF with or without glaucoma and with or without pterygium among the cataract patients are the inclusion criteria.

Exclusion criteria

Patients with presenile cataracts, complicated glaucoma, and pseudo-pterygium and patients who have stayed in this region for less than six months are the exclusion criteria.

Study population and data collection

The records of 352 patients who attended a semi-arid district tertiary care referral teaching institute between December 2020 and August 2022 were included based on inclusion and exclusion criteria. Age, gender, occupation, and other demographic details of cataract patients who underwent surgery during the period were collected. Patients' ocular examination findings were extracted, and the presence or absence of PXF and pterygium was noted. Pupillary ruff abnormalities and PXF over the iris, zonules, and lens were selected, and gonioscopic findings were known to see PXF in angles. Particularly about the pterygium, nasal or temporal, and the type of the pterygium was recorded.

Statistical analysis

Data were entered into MS Office 2019, and descriptive analysis was carried out by calculating the mean for continuous variables and the frequency and proportion for categorical variables. A chi-square test was done to determine the association between pterygium and PXF, and a p-value <0.05 is considered statistically significant.

Ethics approval and confidentiality

The Institutional Ethics Review Committee of Sri Devaraj Urs Medical College approved this study under approval number SDUMC/KLR/IEC/475/2022-2023. The patient's identity was concealed, and the confidentiality of data was ensured during the study.

## Results

This retrospective study included 352 patients. Of the 352 patients, 184 (52.27%) were males, and the rest were females, with the mean age being 67.84 ± 13.08 years. Data show that the majority of patients (95%) were agricultural laborers exposed to sunlight and dust for more than six hours a day, as shown in Table 1.

**Table 1:** Exposure to sunlight in patients Total patients (N) = 352

Exposure to sunlight and dust	Number	Percentage
Less than six hours	17	4.83%
Six to eight hours	252	71.59%
More than eight hours	83	23.58%

The proportions of PXF and pterygium were 28.40% and 56.53% among cataract patients, respectively.

In this study, most patients presented with pterygium in the nasal aspect of the eyes rather than temporal; some were progressive, while others were atrophic type. Of the 100 PXF patients, 80% presented with whitish flaky material over the iris, lens, and zonules with atrophied pupillary sphincter, while 20% were subclinical with only pupillary ruff abnormalities [14]. Age and gender distribution of PXF patients is depicted in Table 2.

**Table 2:** Age and gender distribution of pseudoexfoliation patients Total patients = 100

Variable	Males		Females		Total (N)
	N	%	N	%	
61-70 years	14	14%	8	8%	22
71-80 years	32	32%	23	23%	55
81-90 years	13	13%	10	10%	23
Pterygium	27	27%	20	20%	47
No pterygium	33	33%	20	20%	53

Most patients with PXF belonged to the 71-80 age-group, and their mean age was 75.53 ± 6.26 years. Out of 100 patients with PXF, 47 presented with pterygium. The presence of pterygium is significantly associated with PXF (p < 0.05), as shown in Table 3. This table also shows that 43.18% (152) cataract patients had represented with pterygium alone, 15.05% (53) represented with PXF alone, 13.35% (47) had PXF with pterygium, and 28.4% (100) had no PXF and no pterygium.

**Table 3:** Comparative analysis of the presence of pterygium in pseudoexfoliation patients Total number of patients: 100 *p < 0.05, statistically significant

Variable	Pseudoexfoliation	No pseudoexfoliation	Total	Chi-square value	p-value
Pterygium	47	152	199	5.167	0.023*
No pterygium	53	100	153		
Total	100	252	352		

## Discussion

PXF is an extraocular and intraocular degenerative condition that is the leading cause of blindness due to secondary glaucoma [3]. Even though its presence in the eye leads to hazardous complications, differences in examination techniques, awareness, and diagnostic abilities of individuals may leave this condition undiagnosed [15]. Results of this study depict that the mean age of PXF patients was 75.53 ± 6.26 years, indicating that PXF was mainly identified in the elderly age-group. This age trend was comparatively higher than the other studies [1,15]. At the same time, many studies also support the fact that the risk of PXF increases with age, and a possible explanation for this may be decreased gene expression with the increase in age, which in turn causes PXF [5,6,14,16].

Male preponderance was seen, as shown in Table 2, who spent most of their time outdoors as agricultural laborers, as this is the main occupation of this region; this pattern was similar to other studies [7,8]. Patients who spent more than six hours outdoors were seen to develop PXF, which might be related to increased sun exposure and dust. This was in accordance with the previous studies done in South India [1,4,15,17].

This study recorded 100 patients to have PXF out of 352 patients, i.e., 28.40%, which is a very high proportion when compared with previous studies conducted in south Indian areas [1,15,17]. We also found that out of 352 patients who visited the outpatient department, 199 patients presented with pterygium (56.53%), which is also higher than the previous studies conducted in the South Indian population [13]. This alarming proportion in rural areas of the Kolar district has stirred us to find an association between pterygium, conjunctival degeneration, and PXF. Based on previous studies, pterygium and PXF have a common environmental risk factor, i.e., exposure to sunlight and UV radiations [11,12,17]. The records of 100 PXF patients showed 47 patients having pterygium, which was statistically significant (p = 0.023) by the chi-square test, suggesting a strong association between the two degenerative conditions.

**Figure 1:**
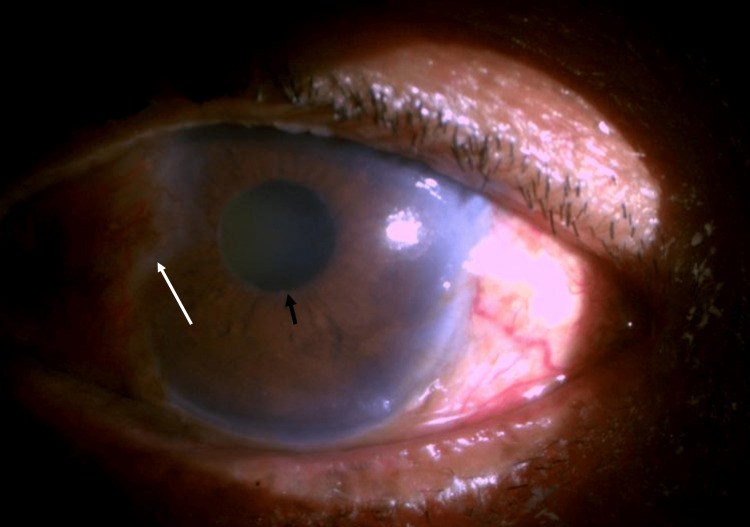
Slit lamp photograph of right eye depicting the presence of nasal pterygium and PXF at pupillary border PXF, pseudoexfoliation The black arrowhead depicts the pseudoexfoliation material on the pupillary border The white arrowhead depicts the progressive nasal pterygium in the right eye

In contrast to this study, Rao et al. reported an association of conjunctival melanosis in early PXF with no association with pterygium [12]. The high number of patients and environmental factors in the present study support the contradicting results of the association between the two degenerative conditions. Figure 1 shows pterygium and PXF in a patient’s right eye.

To the best of the authors’ knowledge, there is a paucity of data regarding this association globally. Also, in India, limited data discuss this association except for the study by Rao et al., which shows a strong association between conjunctival melanosis and PXF [12]. The above association strongly suggests that susceptibility to the development of PXF is high in geographical areas with high exposure to sunlight and UV radiations, along with genetic susceptibility [12]. Further, their role is endorsed by the finding of pterygium in the sun-exposed nasal and temporal areas of the eye [13]. In contrast, many others found no association between conjunctival degenerations and PXF, which again explains the environmental differences between the studies and genetic susceptibility between the two ethnicities [13].

Strengths and limitations

This study is the first to depict a strong association between pterygium and PXF in elderly cataract patients. This study data represents the entire district with a modest sample size. However, as already stated, this study is a one-time association, and further longitudinal data is necessary to validate the above finding. Further detailed histopathological examination of the pterygium is not done to corroborate the finding of PXF in these cases. Also, in this study, only exposure to sun and dust exposure is studied as a risk factor for PXF and pterygium.

## Conclusions

As the conjunctiva is an independent source of exfoliative material in picking the preclinical cases of PXF, with the common precursor being exposure to sunlight and UV radiation, pterygium can be pivotal in predicting the development of PXF in the subclinical or clinically normal-looking eye of unilateral cases. Hence, we would like to draw the attention of ophthalmologists to consider the possibility of developing subclinical PXF in patients with pterygium and to take suitable precautions when subjected to cataract surgery.
